# Comparison of “Huaxi-1” or “histidine-tryptophan-ketoglutarate” cardioplegia in an animal model

**DOI:** 10.3389/fcvm.2024.1385253

**Published:** 2024-06-05

**Authors:** Xiang Yu, Wen Xiong, Jie Zhang, Jing Lin, Bo Wang, Hong Huang, Lei Du, Jiyue Xiong

**Affiliations:** ^1^ Department of Anesthesiology, West China Hospital, Sichuan University, Chengdu, China; ^2^ Department of Anesthesiology, The First Affiliated Hospital of Hunan University of Chinese Medicine, Changsha, Hunan, China; ^3^ Key Laboratory of Transplant Engineering and Immunology, West China Hospital, Sichuan University, Chengdu, China; ^4^ Chengdu Qingshan Likang Pharmaceutical Co. Ltd., Research and Development Department, Chengdu, Sichuan, China

**Keywords:** cardiopulmonary bypass, cardioplegia, histidine-tryptophan-ketoglutarate solution, myocardial protection, animal experiment

## Abstract

**Background:**

Using a pig model of cardiopulmonary bypass, we compared outcomes after cardioplegia either with our in-house “Huaxi-1” solution containing natural blood and crystalloid or with the entirely crystalloid, commercially available “histidine-tryptophan-ketoglutarate” solution.

**Methods:**

Cardiopulmonary bypass was established in 13 healthy male pigs. Twelve of those animals were randomized to receive a single dose of either Huaxi-1 or entirely crystalloid cardioplegia, while the remaining animal was assigned to receive Huaxi-1 without randomization. All animals were then subjected to whole-heart ischemia for 90 min, followed by 2 h of reperfusion, after which myocardial injury was assessed in terms of cardiac function, myocardial pathology and levels of biomarkers in plasma, while levels of high-energy phosphate in myocardium were assayed using liquid chromatography.

**Results:**

Animals given Huaxi-1 cardioplegia required significantly less time to be weaned off bypass, they received significantly lower doses of norepinephrine, and they showed significantly higher levels (mean ± SD) of adenosine triphosphate (14 ± 4 vs. 8 ± 2 µg/mg, *P *= 0.005), adenosine diphosphate (16 ± 2 vs. 13 ± 2 µg/mg, *P *= 0.046), and total adenine nucleotide (37 ± 4 vs. 30 ± 3 µg/mg, *P *= 0.006) in myocardium after 2 h of reperfusion. They also showed less severe bleeding, edema and injury to mitochondria and myofibers in myocardium. The two groups did not differ significantly in doses of inotropic drugs received, cardiac output or levels of biomarkers in plasma.

**Conclusions:**

In this animal model of healthy hearts subjected to 90 min of ischemia, Huaxi-1 cardioplegia may be superior to entirely crystalloid cardioplegia for promoting energy generation and attenuating ischemia/reperfusion injury in myocardium.

## Introduction

Stopping the heart during cardiac surgery is necessary in order to maintain a clear surgical field, but it exposes the heart to ischemia/reperfusion injury. To compensate for this, patients are routinely given cardioplegia solution composed either of a mixture of human blood and crystalloid or of only crystalloid (1–3). Cardioplegia solution is meant to provide a high concentration of metabolic substrates in order to protect the myocardium. Adding blood to the crystalloid can also provide oxygen and potassium.

Several cardioplegia solutions have been commercialized in different countries and are widely used, but which formulations lead to better prognosis is difficult to predict in advance. Also unclear is whether, and under which conditions, blood-crystalloid cardioplegia leads to better outcomes than crystalloid-only cardioplegia. Chinese hospitals typically prepare cardioplegia solutions in-house according to internal practices, or they purchase the entirely crystalloid cardioplegia solution “histidine-tryptophan-ketoglutarate” (HTK) (4), which is the only cardioplegia formulation commercially available in the country and is widely used in other countries (5, 6). HTK cardioplegia can effectively protect the myocardium by reducing energy metabolism (1, 7). Nevertheless, our medical center, where more than 2,000 cardiac surgeries are performed each year, routinely uses an in-house formulation called “Huaxi-1” cardioplegia solution, which was developed in 2007 by mixing blood and custom-designed crystalloid. Though it has not yet been approved by the China Food and Drug Administration, Huaxi-1 cardioplegia has consistently given satisfactory results since its implementation.

We wondered how outcomes after cardiac surgery with this in-house formulation compared to outcomes after use of HTK. Therefore we compared the two cardioplegia solutions in a pig model of cardiopulmonary bypass involving healthy hearts.

## Material and methods

Animal procedures were approved by the Institutional Animal Care Committee of West China Hospital, Sichuan University (2021011A) and conducted in compliance with the “Guidelines for Animal Experimentation” of Sichuan University.

### Animals and cardioplegia solutions

Fourteen male pigs approximately 4 months old and weighing 53.7 ± 3.1 kg (Dashuo Laboratory Animals, Chengdu, China) were housed for one week at the Laboratory Animal Center of West China Hospital, then fasted for 12 h with *ad libitum* access to water immediately before the surgical procedures. One was exited from the study because of coronary artery injury during thoracotomy. Twelve animals were randomized 1:1 according to sealed, opaque envelopes to receive one of two cardioplegia solutions. An additional animal was assigned to the Huaxi-1 group without randomization. Female pigs were not used in the study because estrogen may exert cardioprotective effects that might have confounded our analysis (8).

Cardioplegia solutions were obtained as ready-to-use HTK crystalloid (Koehler Chemie, Alsbach-Hähnline, Germany) or prepared in-house by mixing pig blood with commercial crystalloid (Qingshan Likang, Chengdu, China) in a 4:1 (blood: crystalloid) ratio. The crystalloid contains the following components per 1,000 ml: sodium chloride (7.3 g), mannitol (13.29 g), magnesium sulfate (8.3 g), lidocaine hydrochloride (0.57 g), glucose injection (2.52 g), sodium bicarbonate (5.0 g), and insulin (12 Units). The compositions of the two solutions are shown in Table 1. The cardioplegia solutions were prepared and labelled before surgery by a researcher who was not involved in allocating animals to groups or in analyzing their tissues after surgery.

**Table 1:** Composition of the crystalloid components of the two cardioplegia solutions in this study.

Ion or molecule	Concentration, mmol/L	
	Huaxi-1 solution^a^	HTK solution
K^+^	20.77 (4.5)	9
Mg^2+^	6.88 (0.9)	4
Na^+^	146 (140)	15
Ca^2+^	0.25 (1.24)	0.02
HCO_3_^¯^	31.1 (25)	0
Cl^¯^	120.94 (102)	100
SO_4_^2–^	7.68 (2)	0
Mannitol	13.17	29.97
Glucose	6.52 (5)	0
Lidocaine	0.35	0
Histidine	0	198
Tryptophan	0	2
Ketoglutarate	0	1

HTK, Histidine-tryptophan-ketoglutarate.

^a^ Concentrations of ions or molecules in the blood component of this solution are indicated in parentheses.

### Procedures prior to cardiopulmonary bypass

Animals were administered Zoletil (100 mg; Virbac, Carros, France) and atropine (0.5 mg; Sunnyhope, Chengdu, China) intramuscularly, then midazolam (5 mg/kg; NHWA, Xuzhou, Chnia) and propofol (4 mg/kg; 1% Disoprivan, Corden Pharma, Caponago, ltaly) intravenously. Animals were intubated and mechanically ventilated with a volume-controlled ventilator (Datex-Ohmeda Excel 210, Soma Technology, Cheshire, CT, USA) at an inspired oxygen fraction of 0.4 and respiratory frequency of 16–18 per minute. Throughout the procedure, tidal volume was adjusted to maintain arterial partial pressures of CO_2_ at 35–45 mmHg and O_2_ above 100 mmHg. Anesthesia was maintained through continuous intravenous infusion of propofol (60–120 mcg/kg per min) and midazolam (0.1 mcg/kg per h) as well as vecuronium bromide (0.2 mg/kg per h) to relax muscles. When necessary, animals received 1% isoflurane by inhalation.

Throughout the procedure, PICCO monitoring was performed (Pulsion, Feldkirchen, Germany) via a 7-F catheter (Scw Medicath, Shenzhen, China) inserted into the internal jugular vein. Cardiac output was measured continuously with the PICCO monitor, and the left ventricular ejection fraction was determined before bypass using an EPIQ Elite echocardiograph (Philips, Amsterdam, Netherlands) and the Teichholz method (9).

Arterial blood pressure was monitored continuously via a 20-G arterial catheter (BD Bioscience, Sussex, NJ, USA) that had been inserted into the right femoral artery under ultrasound guidance.

Nasopharyngeal and venous blood temperatures were measured continuously using a thermistor integrated into the cardiopulmonary bypass circuit. Urinary output was measured regularly via a 14-F bladder catheter. Body temperature was maintained using a heating blanket. Hydration was maintained by administering Ringer's lactate solution at 10–30 ml/kg per h.

### Cardiopulmonary bypass, cardioplegia, and whole-heart ischemia

Sternotomy was performed to expose the heart. A bolus of heparin (375 U/kg) was injected intravenously, which prolonged activated clotting time beyond 480 s as determined using an ACT-II system (Medtronic, Minneapolis, MN, USA). Arterial perfusion occurred via a 20-F ascending aortic cannula, while venous drainage occurred via a 28-F right atrial cannula (Medtronic).

The cardiopulmonary bypass circuit comprised a roller pump (Maquet, Rastatt, Germany), a Fusion membrane oxygenator (Medtronic) and, when necessary, a hemoconcentrator (Sorin, Mirandola, Italy). The circuit was primed with 750 ml of succinylated gelatin (B. Braun, Melsungen, Germany) and 250 ml of mannitol. Bypass was initiated with blood flow at 70 ml/kg, and the aorta was cross-clamped to allow delivery of HTK or Huaxi-1 cardioplegia solution. All animals received cardioplegia solution at 4–8°C for 5 min at a pressure of 150 mmHg in the antegrade direction from the aortic root using an XJ-40-20 delivery system (Xijing Medical Appliances, Xi'an, China).

During bypass, nasopharyngeal temperature was approximately 34°C, and mean arterial blood pressure was maintained at 60–80 mmHg. The left ventricle was decompressed during heart arrest via a venting catheter (Medtronic). During ischemia, ice was placed inside the pericardium to cool the heart. Cardioplegia was repeated for another 5 min if the electrocardiograph detected cardiac activity.

Whole-heart ischemia was performed for 90 min, which is the maximal typical duration in Chinese cardiac centers using HTK cardioplegia. Then the cross-clamp was removed to allow reperfusion for 120 min. If ventricular fibrillation occurred, lidocaine (1 mg/kg) and amiodarone (150 mg) were administered; if fibrillation persisted, electroshocks were applied. The left ventricular ejection fraction was determined at 30, 60 and 120 min after declamping.

Animals were weaned off bypass if they showed nasopharyngeal temperature of 37°C, systolic blood pressure >90 mmHg, diastolic blood pressure of 50–75 mmHg, heart rate of 70–120 beats/min, arterial partial O_2_ pressure >100 mmHg, and normal electrolyte levels. Animals could receive inotropic support comprising epinephrine, norepinephrine, milrinone and vasopressin if necessary in order to maintain the mean arterial pressure above 60 mmHg and the cardiac index above 2 L/min per m^2^. The decision to provide inotropic support was based on the animal's vasoactive-inotropic score (10). Weaning was considered a failure if hemodynamics did not stabilize within 2 h on cardiopulmonary bypass.

All animals received protamine in a 1:1 ratio to heparin, after which all cannulae were removed.

### Analysis of blood

Blood samples were harvested from the central vein before surgery, after 85 min of ischemia, and after 30, 60, and 120 min after reperfusion. Samples were centrifuged at 4°C, and the plasma was assayed against creatine kinase isoenzyme, cardiac troponin I, cardiac troponin T, brain natriuretic peptide and lactic dehydrogenase using enzyme-linked immunosorbent assays from LSBio (Seattle, WA, USA). Investigators who sampled and analyzed blood were blinded to which type of cardioplegia solution each animal received.

### Analysis of myocardial tissue

At the end of 2-h reperfusion, animals were euthanized using an overdose of potassium, then myocardial samples of the left ventricular apex were harvested and stored in liquid nitrogen for subsequent assay for levels of high-energy phosphate in myocardium, or fixed in 4% paraformaldehyde for subsequent histology, or fixed in 2.5% glutaraldehyde for subsequent electron microscopy. Investigators who prepared and analyzed these tissues were blinded to which type of cardioplegia solution each animal received.

For assay of high-energy phosphate, myocardial tissue (100 mg) was added to 1 ml of ATP buffer (catalog no. BC0304, Solarbio, Beijing, China), mechanically homogenized using a SCIENTZ-48 system (Ningbo Scientz Biotechnology, Ningbo, China), and centrifuged at 13,000 g for 10 min at 4°C. Equal amounts of protein for each sample were mixed with 5% perchloric acid, centrifuged again at 20,000 g for 15 min at 4°C, and the resulting protein-free supernatant was neutralized with 3 M K_2_CO_3_ (pH 6.5–6.7).

Supernatant was analyzed using a Series 1,100 HPLC System (Agilent Technologies, Santa Clara, CA, USA) with detection at 262 nm. Chromatographic separation was performed on a Chromplus C18 column (5 µm, 250 × 4.6 mm; Swell, Chengdu, China) at 25°C. The mobile phase consisted of 6 mmol/L tetrabutylammonium hydroxide in 0.05 mol/L phosphate buffer (pH 6.0) (A) and acetonitrile (B) in the A:B ratio 91:9. Supernatant (10 µl) was injected and eluted at a flow rate of 1 ml/min.

For histology, myocardial tissue was fixed overnight in 4% paraformaldehyde solution and dehydrated through an ethanol gradient. Then samples were paraffin-embedded and cut into 4-μm sections for staining with hematoxylin-eosin. Sections were examined at 40× magnification under a bright field microscope (Zeiss, Jena, Germany). Sections were graded based on the extent of bleeding, edema, damage to myocardial fibers and infiltration by inflammatory cells. The severity of these four features was graded as not obvious (0 point), slight (1 point), moderate (2 points), extensive (3 points) or severe (4 points) (11). Scores were averaged from five tissue sections per animal in order to obtain the final score for each animal.

For electron microscopy, sections were prepared and analyzed under an HT7800 transmission electron microscope (Hitachi, Tokyo, Japan). Abnormal ultrastructure of nuclei, mitochondria and myofibers was categorized as described previously (12–14). In brief, nuclei were scored as (1) if the nuclear membrane was intact, nuclear ultrastructure was clear, and the nucleolus was obvious; (2) if the nuclear membrane was wrinkled, heterochromatin had accumulated around the perimeter of the nuclear membrane, and the nucleolus was visible; or (3) if the nuclear membrane had dissolved and the nucleolus was unclear. Mitochondria were scored as 0 if they appeared normal; (1) if their overall structure seemed normal but they lacked matrix granules; (2) if they were swollen and the matrix was clear; (3) if they were swollen, the matrix was clear, and they showed ridge fracture or matrix fusion; or (4) if they showed the same features as for 3 points as well as destruction of inner and outer membranes. Myofibers were scored as (1) if the myofibrillar cleft was ordered and the myotome clear; (2) if they showed some fusion and the myotome was unclear; or (3) if they showed swelling, disorder or fracture and the myotome was unclear. These scores were averaged for three randomly selected fields of view at 8,000× per animal.

## Statistical analysis

Data were analyzed using SPSS 26 (IBM, Chicago, IL, USA), and GraphPad Prism 9 (GraphPad, Boston, MA, USA) was used to prepare data plots. Categorical data were reported as *n* (%), and intergroup differences were assessed for significance using Fisher's exact probability method. Continuous data were reported as mean ± standard deviation or median (interquartile range), and intergroup differences were assessed using the independent-samples *t*-test if the data were normally distributed, or using the Wilcoxon test otherwise. Differences associated with *P* < 0.05 were considered significant.

## Results

Here we compared our in-house blood cardioplegia product Huaxi-1 to HTK cardioplegia, which lacks glucose and insulin that can promote energy generation in the myocardium (Table 1). Of the fourteen animals initially included, one was exited from the study because of coronary artery injury during thoracotomy. The remaining animals were included in the final analysis of the Huaxi-1 group (n = 7) and HTK group (n = 6). The Huaxi-1 and HTK groups did not differ significantly in terms of pig weight before the procedure (52.5 ± 3.6 kg vs. 55 ± 1.8 kg, *P *= 0.16), perfusion pressure (147 ± 11 mmHg vs. 150 ± 6 mmHg, *P *= 0.58), or use of lidocaine, amiodarone or defibrillation to restore sinus rhythm (data not shown). Two animals in the HTK group and one in the Huaxi-1 group were administered amiodarone because of defibrillation after cross-clamp removal. None of the animals showed electrocardiographic activity during the 90-min ischemia, so all animals received only one 5-min dose of their assigned cardioplegia solution. All animals were successfully weaned off cardiopulmonary bypass, which occurred within 30 min after the start of reperfusion in all but one animal in the HTK group.

The Huaxi-1 group received significantly smaller volume of cardioplegia solution [1,250 (1,250, 1,250) vs. 1,550 (1,500, 1,713) ml, *P *= 0.001], experienced cardiac arrest significantly sooner after the start of cardioplegia delivery (28 ± 7 vs. 78 ± 52 s, *P *= 0.028), and was weaned off bypass significantly sooner after the start of reperfusion (13 ± 9 vs. 29 ± 14 min, *P *= 0.039).

The two groups did not differ significantly in hemodynamics (Table 2) or requirement for inotropic drugs (Table 3), except that a significantly lower dose of norepinephrine was given to the Huaxi-1 group.

**Table 2:** Comparison of hemodynamic parameters in the two groups of animals.

Parameter	Minutes after reperfusion start^a^	Cardioplegia solution		*P*
		Huaxi-1	HTK	
Systolic blood pressure (mmHg)	0	100 ± 11	101 ± 12	0.85
	30	104 ± 6	103 ± 12^b^	0.79
	60	99 ± 15	100 ± 14	0.91
	120	105 ± 18	97 ± 13	0.38
Diastolic blood pressure (mmHg)	0	55 ± 10	57 ± 13	0.74
	30	55 ± 9	48 ± 9^b^	0.16
	60	50 ± 7	48 ± 15	0.72
	120	51 ± 11	49 ± 9	0.66
Mean arterial pressure (mmHg)	0	71 ± 9	73 ± 14	0.79
	30	74 ± 7	67 ± 9^b^	0.17
	60	69 ± 10	66 ± 14	0.63
	120	72 ± 14	68 ± 7	0.54
Ejection fraction (%)	0	61 ± 1	61 ± 1	0.97
	30	61 ± 3	59 ± 1^b^	0.15
	60	61 ± 2	60 ± 2	0.58
	120	61 ± 2	60 ± 1	0.97
Cardiac output (L/min)	0	4.08 ± 0.65	4.25 ± 0.71	0.65
	30	4.38 ± 0.92	4.02 ± 0.87^b^	0.52
	60	4.72 ± 0.76	4.21 ± 0.61	0.21
	120	4.96 ± 0.63	5.0 ± 0.88	0.91

Values are mean ± SD, unless otherwise noted. HTK, histidine-tryptophan-ketoglutarate.

^a^ The “0” time point refers to before bypass.

^b^ Data are from five of the six animals because one animal could not be weaned from bypass within 30 min after the start of reperfusion.

**Table 3:** Comparison of vasoactive drugs given to the two groups of animals.

Parameter	Minutes after reperfusion start	Cardioplegia solution		*P*
		Huaxi-1	HTK	
Epinephrine dose, mcg/min per kg	30	0.02 [0.00, 0.03]	0.06 [0.01,0.18]	0.15
	60	0.01 [0.00, 0.03]	0.03 [0.01,0.05]	0.15
	120	0.01 [0.00, 0.03]	0.03 [0.01,0.05]	0.24
Animals receiving epinephrine	30	4 (57.1)	6 (100)	0.19
	60	4 (57.1)	6 (100)	0.19
	120	4 (57.1)	5 (85.5)	0.56
Norepinephrine dose, mcg/min per kg	30	0.00 [0.00, 0.00]	0.00 [0.00,0.00]	0.82
	60	0.00 [0.00, 0.00]	0.01 [0.00,0.03]	0.12
	120	0.00 [0.00, 0.00]	0.04 [0.00,0.05]	0.03
Animals receiving norepinephrine	30	1 (14.3)	1 (16.7)	1.00
	60	1 (14.3)	3 (50)	0.27
	120	1 (14.3)	4 (66.7)	0.10
Maximum VIS score	30	1.86 ± 0.7	9.33 ± 3.61	0.09
	60	1.57 ± 0.61	5.33 ± 1.54	0.06
	120	1.57 ± 0.61	6.33 ± 2.32	0.09

Values are *n* (%) or median [interquartile range], unless otherwise noted. VIS, the vasoactive inotropic score.

**Figure 1:**
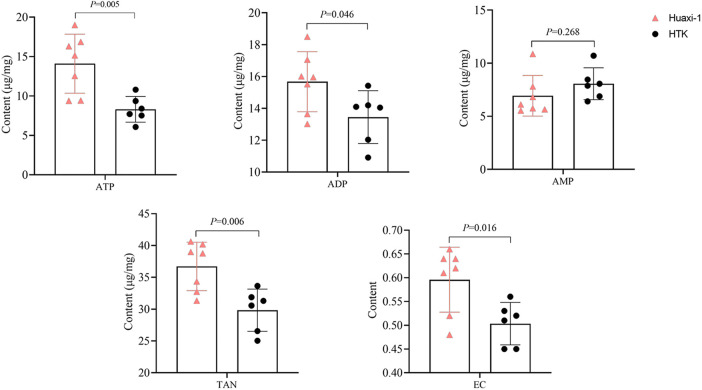
Levels of high-energy phosphate and other energy indices in left ventricular apical myocardium in each animal group after 2 h of reperfusion. ADP, adenosine diphosphate; AMP, adenosine monophosphate; ATP, adenosine triphosphate; EC, energy charge; HTK, histidine-tryptophan-ketoglutarate; TAN, total adenine nucleotide.

At the end of the experiment, the apical myocardium in the left ventricle of Huaxi-1 animals contained significantly higher levels of adenosine triphosphate (14 ± 4 vs. 8 ± 2 µg/mg) and adenosine diphosphate (16 ± 2 vs. 13 ± 2 µg/mg; Figure 1). As a result, the same tissue from Huaxi-1 animals also showed significantly higher levels of total adenine nucleotide (36.7 ± 3.8 vs. 29.8 ± 3.3 µg/mg) and energy charge (0.6 ± 0.07 vs. 0.5 ± 0.04). These results suggest that Huaxi-1 cardioplegia provided greater energy to the myocardium.

**Figure 2:**
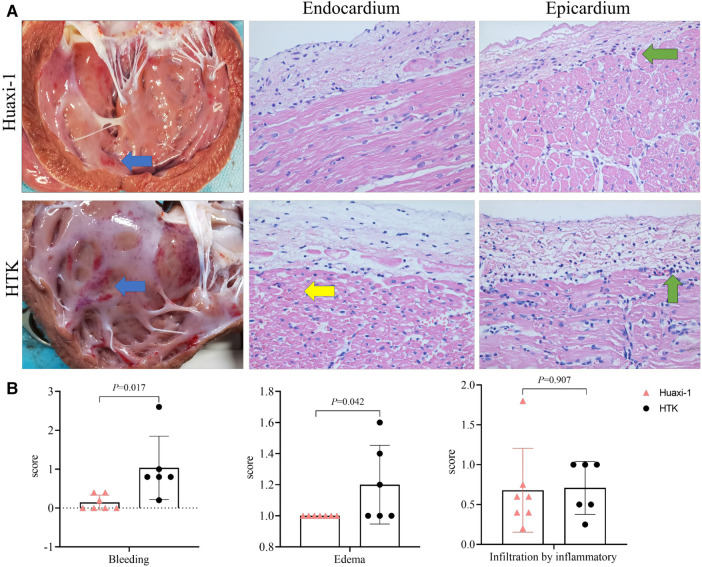
Comparison of myocardial pathology between the two groups of animals at the end of surgery. (**A**) Histopathology of thin sections of heart after staining with hematoxylin-eosin. The *left* column shows representative photographs of the myocardium, in which blue arrows indicate sub-endocardial bleeding. The *middle* column shows representative micrographs of endocardium, in which the yellow arrow indicates microcirculatory bleeding in the sub-endocardium. The *right* column shows representative micrographs of epicardium, in which green arrows indicate infiltration by inflammatory cells. Magnification, 40×. (**B**) Histopathology scores for bleeding, edema and infiltration by inflammatory cells. HTK, histidine-tryptophan-ketoglutarate.

**Figure 3:**
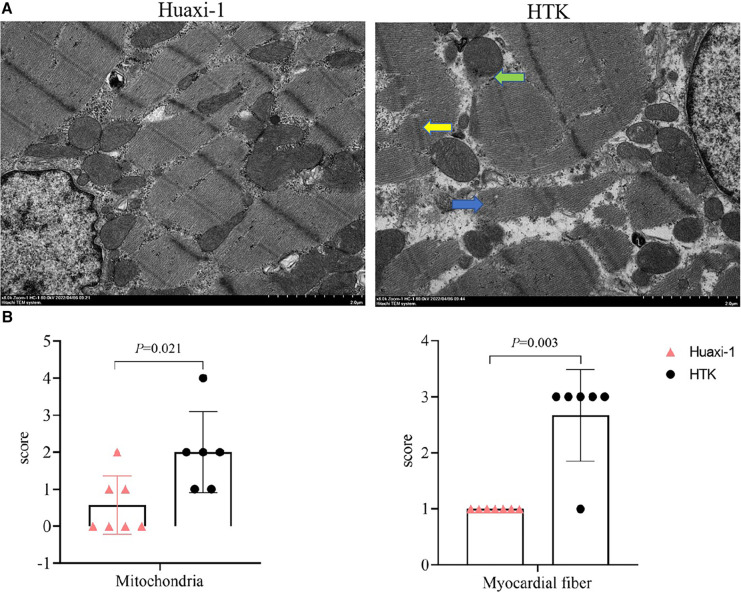
Transmission electron micrographs of myocardial tissue from the two groups of animals at the end of surgery. (**A**) Representative micrographs are shown. The green arrow indicates mitochondrial membrane rupture; the blue arrow, disruption of myocardial fibers; and the yellow arrow, distortion of the Z line with partial expansion of intercalated discs and sarcoplasmic reticulum. Scale bar, 2.0 μm. (**B**) Scores for severity of pathology observed in mitochondria and myocardial fibers. HTK, histidine-tryptophan-ketoglutarate.

Myocardial endocardium after 2 h of reperfusion showed petechial hemorrhaging but was intact in Huaxi-1 animals, whereas it showed numerous hemorrhagic patches in HTK animals, leading to significantly lower histopathology scores for bleeding and edema in Huaxi-1 animals (Figure 2). Tissue from the two groups showed similar levels of endocardial infiltration by inflammatory cells. Electron microscopy revealed significantly less damage to mitochondria and myofibers in the myocardium of Huaxi-1 animals (Figure 3). Neither group showed obvious damage to nuclear ultrastructure. These results suggest that Huaxi-1 cardioplegia was associated with less cellular and subcellular damage to the myocardium.

**Figure 4:**
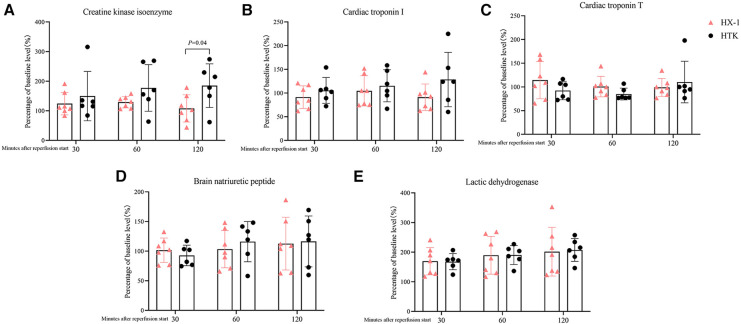
Comparison of levels of myocardial injury markers in plasma at different times after surgery between the two groups of animals. (**A**) Creatine kinase isoenzyme. (**B**) Cardiac troponin I. (**C**) Cardiac troponin T. (**D**) Brain natriuretic peptide. (**E**) Lactic dehydrogenase. HX-1, Huaxi-1; HTK, histidine-tryptophan-ketoglutarate.

**Figure 5:**
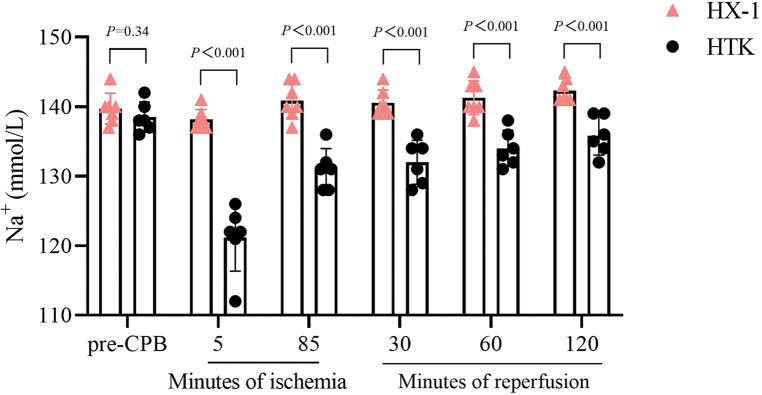
Comparison of sodium ion concentration in serum at different times during surgery between the two groups of animals. HX-1, Huaxi-1; HTK, histidine-tryptophan-ketoglutarate.

All the measured markers of myocardial injury in plasma were similar between the two groups of animals after 2 h of reperfusion, except that the Huaxi-1 group showed significantly lower levels of creatine kinase isoenzyme (Figure 4). Levels of sodium ion in plasma remained normal in Huaxi-1 animals throughout the procedure, whereas sodium levels fell significantly after HTK cardioplegia (138 ± 2 vs. 121 ± 5 mmol/L, *P *< 0.01) and remained low through the end of the procedure (Figure 5). These results suggest that Huaxi-1 cardioplegia was more effective at maintaining normal electrolyte levels in plasma.

## Discussion

Our study with a pig model of cardiopulmonary bypass involving healthy hearts suggests that the combination of blood and crystalloid in Huaxi-1 cardioplegia solution leads to higher concentrations of high-energy phosphates in myocardium, faster recovery of cardiac function, less myocardial injury, and stabler concentrations of sodium and potassium ions in plasma than the entirely crystalloid HTK cardioplegia solution. Even so, a single dose of either type of cardioplegia led to recovery from 90-min whole-heart ischemia in all animals.

The two types of cardioplegia solution reflect different approaches to improving myocardial energy production during heart arrest. During myocardial ischemia, glucose is favored as an energy source and aerobic metabolism is favored over glycolysis, reducing the reliance on free fatty acids that normally occurs in the presence of adequate oxygen (15). In Huaxi-1 cardioplegia, the blood provides substantial oxygen, while the crystalloid provides magnesium ions, glucose and insulin to promote glucose uptake, thereby promoting glycolysis. In parallel, the natural buffers in the blood as well as the sodium bicarbonate in the crystalloid help to neutralize lactic acid and provide an adequate pH environment for glycolytic enzymes. HTK cardioplegia solution, in contrast, carries little oxygen, but it provides α-ketoglutarate and tryptophan to promote glycolysis. It also contains histidine buffer to provide a suitable pH environment (16). Our results suggest that inclusion of blood in cardioplegia solution may lead to higher levels of high-energy phosphates in myocardium.

Huaxi-1 cardioplegia was associated with less injury to mitochondria in our study. Ischemia damages mitochondria by altering the function of Na ^+ ^-K^+^ and Na ^+ ^-H^+^ pumps (17), by triggering Ca^2+^ overload (18), and by promoting the production of reactive oxygen species (19–21). Huaxi-1 was associated with less microcirculatory bleeding and myocardial edema, which may reflect that the much lower Na^+^ concentration in HTK cardioplegia solution damages endothelial cells (22, 23); indeed, pigs treated with this solution showed hyponatremia through the end of the procedure. Myocardial edema can stiffen the ventricular wall and thereby impair cardiac function (24). Although the two types of cardioplegia solution in our study led to similar levels of cardiac function based on echocardiography, Huaxi-1 solution was associated with milder myocardial injury and faster recovery of cardiac function, as indicated by shorter weaning time, lower levels of creatine kinase isoenzyme in plasma, and lower requirement for inotropic norepinephrine.

Our finding that mixed blood-crystalloid cardioplegia led to better outcomes than entirely crystalloid cardioplegia is consistent with studies that have associated HTK cardioplegia with lower cardiac index and ventricular function soon after surgery (25) and higher incidence in spontaneous ventricular fibrillation (26) than cold blood cardioplegia. Our network meta-analysis comparing several types of cardioplegia solutions containing only crystalloid or a combination of crystalloid and blood linked HTK cardioplegia solution to the highest risk of mortality (27). This evidence base argues for including a substantial component of natural blood in cardioplegia solutions.

Our results should be interpreted with caution given that we used only male pigs with healthy hearts. While a previous comparison of cardioplegia solutions also involved only animals with healthy hearts (28), future studies may wish to use animals of both sexes with comorbidities (29, 30) in order to explore the generalizability of our findings to a broad range of patients, especially those with cardiac pathologies. Using propofol as anesthetic, which mimics routine practice with patients at our hospital, may have confounded our analysis because it may exert cardioprotective effects (31). Nevertheless, any confounding should have been balanced between the two groups because both received the same propofol dose. Similarly, amiodarone, which we gave to animals showing ventricular defibrillation, can exert cardioprotective effects (32), yet its frequency of use did not differ significantly between the two groups. We assessed levels of high-energy phosphate and histopathology in myocardial tissue at one time point, after 2 h of reperfusion. It would be important to compare outcomes at different stages of the bypass procedure, including at the end of ischemia and after longer periods of reperfusion.

Despite these limitations, our study involving healthy pig hearts suggests that combining crystalloids with natural blood may protect the myocardium from 90 min of whole-heart ischemia/reperfusion injury better than crystalloid on its own. The superiority appears to lie at least partly in the ability of blood to provide higher levels of high-energy phosphates to drive cardiac metabolism after arrest, despite the fact that HTK cardioplegia solution contains various amino acids and metabolic substrates.

## Data Availability

The original contributions presented in the study are included in the article/**Supplementary Material**, further inquiries can be directed to the corresponding author.
