# Corrigendum: Comparison of “Huaxi-1” or “histidine-tryptophan-ketoglutarate” cardioplegia in an animal model

**DOI:** 10.3389/fcvm.2024.1450474

**Published:** 2024-07-09

**Authors:** Xiang Yu, Wen Xiong, Jie Zhang, Jing Lin, Bo Wang, Hong Huang, Lei Du, Jiyue Xiong

**Affiliations:** ^1^ Department of Anesthesiology, West China Hospital, Sichuan University, Chengdu, China; ^2^ Department of Anesthesiology, The First Affiliated Hospital of Hunan University of Chinese Medicine, Changsha, Hunan, China; ^3^ Key Laboratory of Transplant Engineering and Immunology, West China Hospital, Sichuan University, Chengdu, China; ^4^ Chengdu Qingshan Likang Pharmaceutical Co. Ltd., Research and Development Department, Chengdu, Sichuan, China

**Keywords:** cardiopulmonary bypass, cardioplegia, histidine-tryptophan-ketoglutarate solution, myocardial protection, animal experiment

A Corrigendum on Comparison of “Huaxi-1” or “histidine-tryptophan-ketoglutarate” cardioplegia in an animal model By Yu X, Xiong W, Zhang J, Lin J, Wang B, Huang H, Du L and Xiong J (2024). Front. Cardiovasc. Med. 11:1385253. doi: 10.3389/fcvm.2024.1385253

Data in the Abstract, Results text, Tables 2, 3 and Supplementary Material correspond to analysis of seven animals in the Huaxi-1 group, whereas the text in Methods and the data in Figures 1–5 correspond to analysis of six animals. We deeply regret this discrepancy, which arose through lack of coordination among the authors of the manuscript. The study protocol called for randomizing six animals into the two study arms, so we enrolled 14 animals to ensure six per arm in the final analysis. Of the two extra animals, one was excluded from the study because of coronary artery injury during thoracotomy and the other was arbitrarily assigned to the Huaxi-1 group without randomization.

In the published article, there was an error in Figures 1–5 as published. The corrected figures appear below.

The corrected Figure 1:

**Figure:**
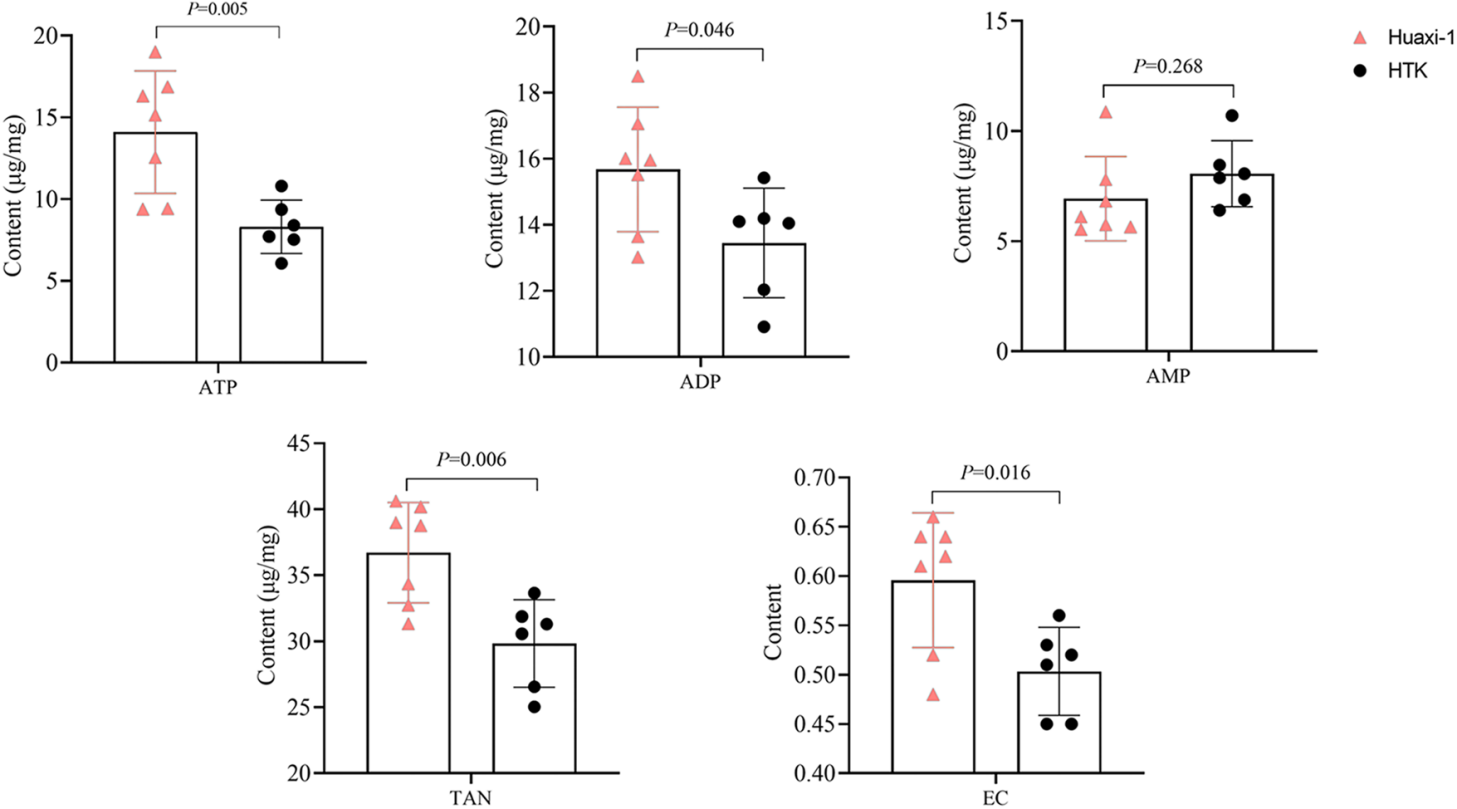


The corrected Figure 2:

**Figure:**
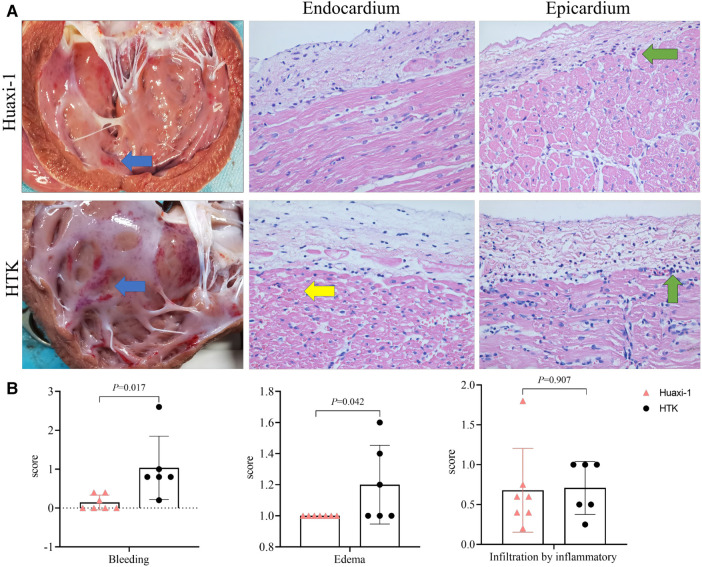


The corrected Figure 3:

**Figure:**
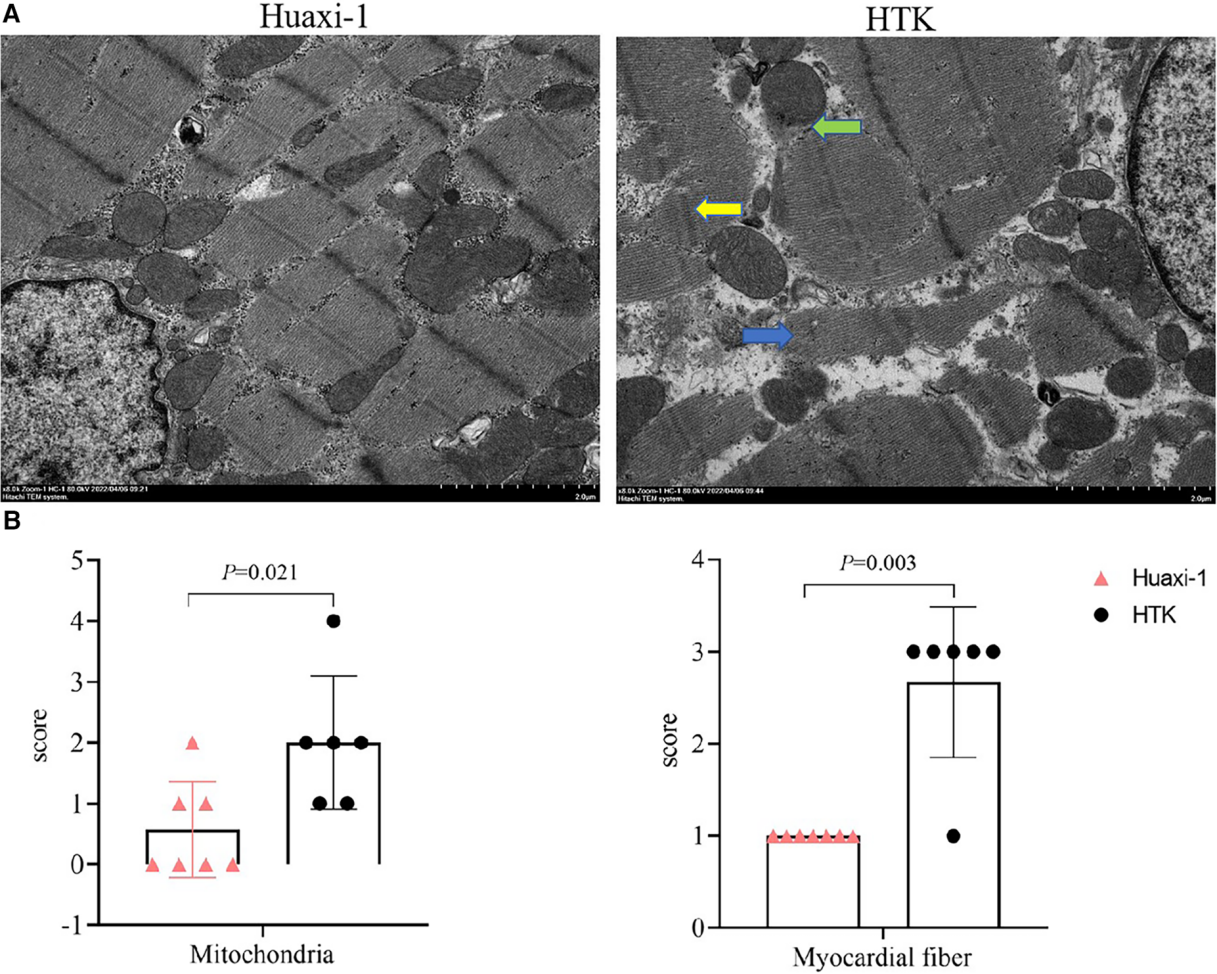


The corrected Figure 4:

**Figure:**
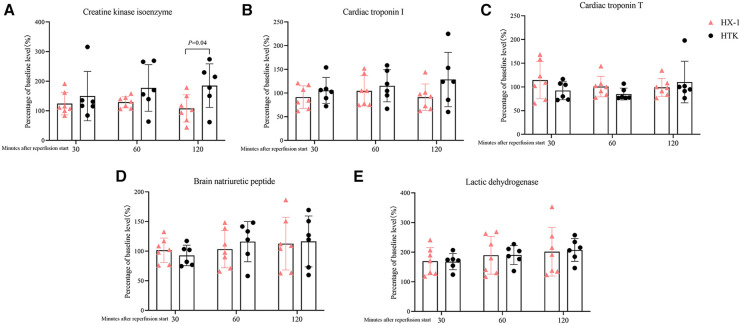


The corrected Figure 5:

**Figure:**
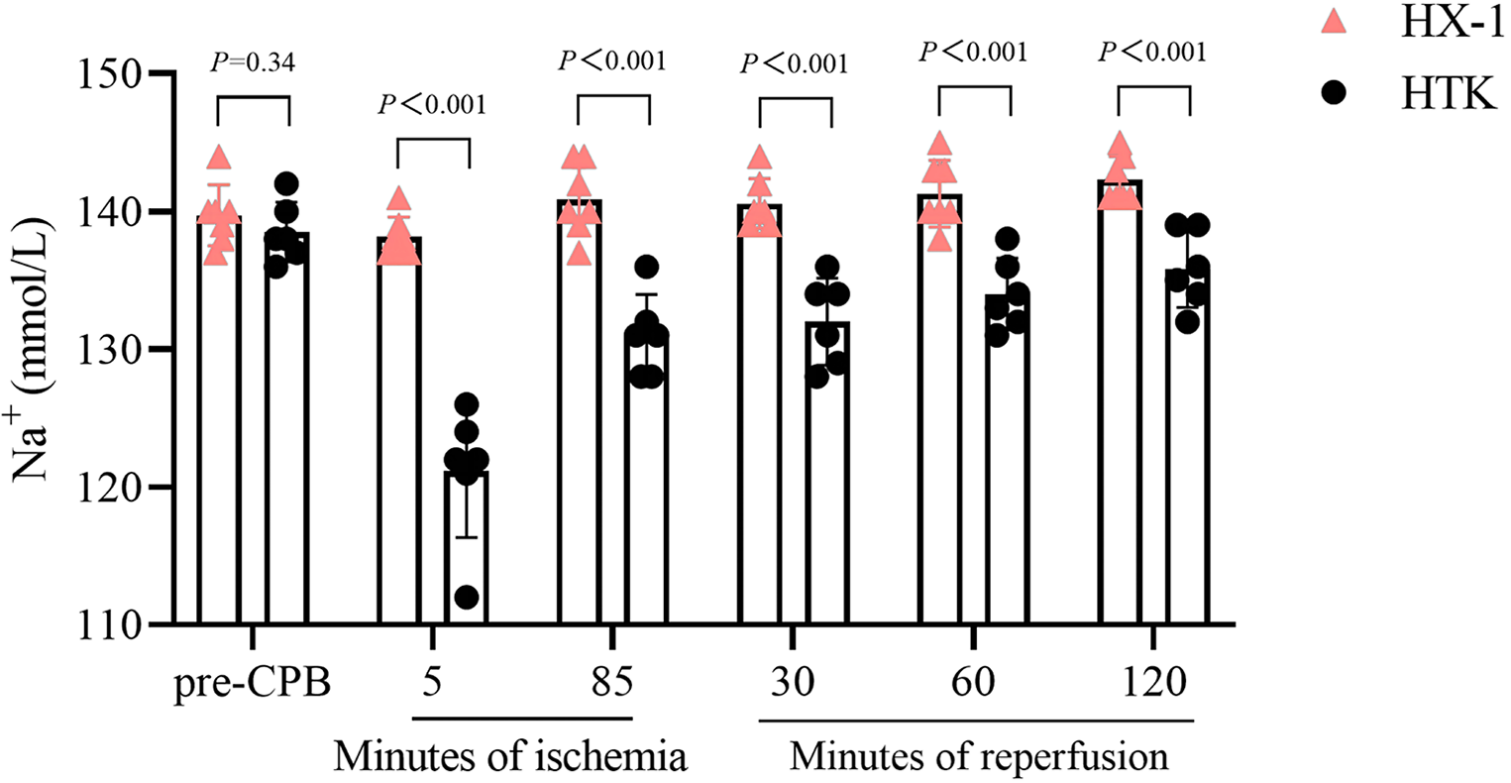


A correction has been made to Abstract, *Methods*. This sentence previously stated:

“Cardiopulmonary bypass was established in 12 healthy male pigs, who were randomized to receive a single dose of either Huaxi-1 or entirely crystalloid.”

The corrected sentence appears below:

“Cardiopulmonary bypass was established in 13 healthy male pigs. Twelve of those animals were randomized to receive a single dose of either Huaxi-1 or entirely crystalloid cardioplegia, while the remaining animal was assigned to receive Huaxi-1 without randomization.”

A correction has been made to Materials and Methods, *Animal and cardioplegia soliutions*, paragraph one. This sentence previously stated:

“Twelve male pigs approximately 4 months old and weighing 53.7 ± 3.1 kg (Dashuo Laboratory Animals, Chengdu, China) were housed for one week at the Laboratory Animal Center of West China Hospital, then fasted for 12 h with *ad libitum* access to water immediately before the surgical procedures. Animals were randomized 1:1 according to sealed, opaque envelopes to receive one of two cardioplegia solutions. Female pigs were not used in the study because estrogen may exert cardioprotective effects that might have confounded our analysis (8).”

The corrected sentence appears below:

“Fourteen male pigs approximately 4 months old and weighing 53.7 ± 3.1 kg (Dashuo Laboratory Animals, Chengdu, China) were housed for one week at the Laboratory Animal Center of West China Hospital, then fasted for 12 h with *ad libitum* access to water immediately before the surgical procedures. One was exited from the study because of coronary artery injury during thoracotomy. Twelve animals were randomized 1:1 according to sealed, opaque envelopes to receive one of two cardioplegia solutions. An additional animal was assigned to the Huaxi-1 group without randomization. Female pigs were not used in the study because estrogen may exert cardioprotective effects that might have confounded our analysis (8).”

A correction has been made to Results, paragraph one. This sentence previously stated:

“Of the thirteen animals initially included, only one had to be excluded before analysis, which was due to injury of the coronary artery during thoracotomy.”

The corrected sentence appears below:

“Of the fourteen animals initially included, one was exited from the study because of coronary artery injury during thoracotomy. The remaining animals were included in the final analysis of the Huaxi-1 group (*n* = 7) and HTK group (*n* = 6).”

The authors apologize for these errors and state that this does not change the scientific conclusions of the article in any way. The original article has been updated.

